# DATSURYOKU Sensor—A Capacitive-Sensor-Based Belt for Predicting Muscle Tension: Preliminary Results

**DOI:** 10.3390/s21196669

**Published:** 2021-10-07

**Authors:** Akihiko Murai, Shusuke Kanazawa, Ko Ayusawa, Sohei Washino, Manabu Yoshida, Masaaki Mochimaru

**Affiliations:** 1 Human Augmentation Research Center, National Institute of Advanced Industrial Science and Technology, Chiba 277-0882, Japan; kanazawa-s@aist.go.jp (S.K.); k.ayusawa@aist.go.jp (K.A.); s.washino@aist.go.jp (S.W.); yoshida-manabu@aist.go.jp (M.Y.); m-mochimaru@aist.go.jp (M.M.); 2 PRESTO, Japan Science and Technology Agency, Saitama 332-0012, Japan

**Keywords:** DATSURYOKU (muscle relaxation), wearable sensing, capacitive sensor, preemptive medicine

## Abstract

Excessive muscle tension is implicitly caused by inactivity or tension in daily activities, and it results in increased joint stiffness and vibration, and thus, poor performance, failure, and injury in sports. Therefore, the routine measurement of muscle tension is important. However, a co-contraction observed in excessive muscle tension cannot be easily detected because it does not appear in motion owing to the counteracting muscle tension, and it cannot be measured by conventional motion capture systems. Therefore, we focused on the physiological characteristics of muscle, that is, the increase in muscle belly cross-sectional area during activity and softening during relaxation. Furthermore, we measured muscle tension, especially co-contraction and relaxation, using a DATSURYOKU sensor, which measures the circumference of the applied part. The experiments showed high interclass correlation between muscle activities and circumference across maximal voluntary co-contractions of the thigh muscles and squats. Moreover, the circumference sensor can measure passive muscle deformation that does not appear in muscle activities. Therefore, the DATSURYOKU sensor showed the potential to routinely measure muscle tension and relaxation, thus avoiding the risk of failure and injury owing to excessive muscle tension and can contribute to the realization of preemptive medicine by measuring daily changes.

## 1. Introduction

The rapid aging of the population and declined birthrates as well as advanced medical technology and increased medical requirements have rapidly increased medical costs and demand for nursing care. In addition, the number of young people in the workforce is reducing. Therefore, protecting the lives of the elderly and their families as well as coping with the rising burden of medical costs are becoming major issues. To address these issues, a new direction of medicine has been proposed as preemptive medicine, which aims to diagnose and predict diseases with a certain probability, and to provide therapeutic intervention in the early stage of disease onset, when there are no clinical symptoms and no abnormalities in the usual examination findings [1]. The above proposal focuses on diseases with genetic predisposition based on biomarkers, such as biochemical findings of protein, RNA, and imaging findings. However, to avoid an increase in demand for nursing care and the burden of medical costs, the motor system for diseases caused by the decline in physical and cognitive functions and daily exercise habits associated with aging (as typified by the decline in walking ability owing to pain and dullness of knee joints, such as knee osteoarthritis) should be improved to enable people to live independently for a longer period and extend their health span. Preemptive medical treatment for diseases related to such motor functions requires diagnosis and prediction in the early stages of disease onset based on changes in the state of cardiopulmonary and musculoskeletal functions as well as daily exercise, and routine measurement of this information is essential.

In this study, we developed a wearable device that realizes measurement of the muscle state, especially excessive muscle tension (co-contraction), as a state of daily motor function, and also realizes muscle relaxation, which is a cognitive avoidance of muscle tension based on biofeedback. Excessive muscle tension is often implicitly caused by inactivity and tension in daily life, leading to increased joint stiffness and vibration, which in turn causes decreased performance in sports, breakdowns, injuries, and defects and diseases in daily activities, such as contractures and tremors. Therefore, the measurement of the muscle state, including excessive muscle tension, is effective for injury prevention and performance improvement [2].

However, the measurement of muscle state in daily life has not yet been realized owing to several technical issues. For example, in commercially available wearable devices, vital sensing techniques, such as the heart rate, respiratory rate, and blood pressure, have been developed by pulse oximeter-based measurement technology [3,4,5,6]. However, muscle state is measured by direct measurement of activity using electromyography (EMG), which is the potential difference between two points along a muscle fiber. This activity should be measured at a high sampling rate, such as 1–2 kHz for the frequency characteristics of EMG signals; thus, this measurement is power consuming. In addition, it is difficult to maintain a stable electrical contact impedance between the body surface and the electrodes for a long period; thus, the daily routine measurement of muscle activity with wearable devices has not yet been realized [7]. Mechanomyography (MMG) is also applied to measure physiological muscle deformation by tensile strength variation of piezoresistive sensors [8,9]. This approach realizes measurement of single muscle contraction, though it is difficult to measure global muscle status, e.g., co-contraction. In indirect measurements, muscle activity is estimated by motion measurement and analysis using a musculoskeletal model [10,11,12]. Here, the joint angles and torques are calculated by solving the kinematics and dynamics of the musculoskeletal model based on motion data measured using an optical motion capture system or an inertial measurement unit (IMU) motion capture system [13] and force plates. The joint torques are then distributed to the muscle activities based on the geometric relationship between the muscles and skeleton. However, a redundancy problem occurs because the number of muscles is significantly larger than the number of degrees of freedom of the skeleton. Mathematical optimizations, such as minimizing the sum of squares of muscle tension, are applied to this optimization; thus, muscle co-contraction, which is redundant, is counteracted.

With this background, this study aims to directly measure muscle states, including excessive muscle tension (co-contraction), in a cross-modal manner. The muscle has several characteristics, based on its physiological structure and mechanism of activity. During activity, the muscle contracts by overlapping actin filaments and myosin filaments, and the cross-sectional area of the muscle belly increases during the activity owing to this physical phenomenon. However, relaxation softens muscle and makes it more susceptible to deformation in response to external forces and inertial forces. We focused on these deformations related to muscle activity and relaxation, and then estimated the muscle state in a cross-modal manner by measuring the circumference of the muscle. In this approach, there is no problem of electrical contact impedance between the body surface and electrode as described above, and no problem of power consumption because the sampling rate of general motion (100–200 Hz, which is often applied in the commercial motion capture systems) is sufficient for the measurement. Furthermore, it is possible to estimate excessive muscle tension owing to direct measurement of the muscle state.

In this study, we developed a DATSURYOKU sensor that realizes a measurement of muscle state that involves tension as well as stable relaxation. The DATSURYOKU sensor is a capacitive sensor in the form of a wearable belt. It includes the dielectric of a silicone elastomer sandwiched between stretchable electrodes prepared by the flocking of silver-plated fibers. It can convert the change in the circumference of the wearing part into a change in capacitance. We validated the estimation of the muscle state by conducting human subject experiments using the DATSURYOKU sensor and conventional measurement devices to measure the muscle state in a multimodal manner. In this experiment, we measured motion using an optical motion capture system, floor reaction force by a force plate, and muscle activity using EMG. The muscle state is measured multimodally during excessive muscle tension (co-contraction) and walking. The analysis results show a high interclass correlation between muscle activity and circumference, and we showed that the DATSURYOKU sensor has the potential to estimate the muscle state that involves tension and relaxation.

This paper is organized as follows: Section 2 describes the features and development of the DATSURYOKU sensor as well as the experiments on human subjects. Section 3 presents the results of the experiments and data analysis. Section 4 discusses the results, and conclusions are presented in Section 5.

## 2. Materials and Methods

In this section, we describe the features and development of the DATSURYOKU sensor, as well as human subject experiments for validation.

### 2.1. Sensor Fabrication

We developed a prototype wearable sensor that can measure the change in the circumference of a human thigh in real time. The sensor was designed to have two conductive layers that can be stretched and shrunk, and a dielectric layer sandwiched between them. The aim of the design was to output the change in length as a change in the capacitance of the dielectric layer. The core of the sensor is a stretchable conductive layer, which is formed by the flocking of silver-plated fibers that have been cut into short lengths. The conductive layer can maintain its conductivity even when stretched, as previously reported by Nobeshima et al. [14].

**Figure 1:**
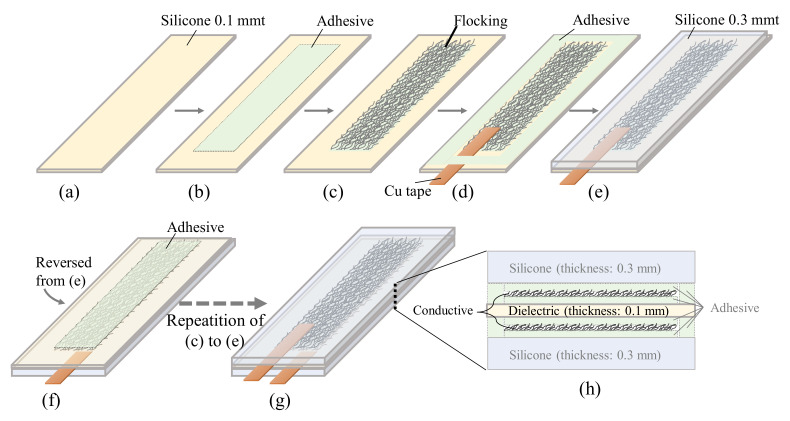
Process flow for fabricating the capacitive-stretch sensor (**a**–**g**) and its schematic cross section (**h**).

The fabrication flow of the DATSURYOKU sensor and a schematic diagram of the cross-section to be fabricated are presented in Figure 1. To prepare the dielectric layer, a silicone sheet (SC50NNS-100, AS ONE, Tokyo, Japan) with a thickness of 0.1 mm was manually cut into 35.0 mm wide and 200 mm depth (a). A double-faced tape (NT-1001-36-0100, AS ONE, Tokyo, Japan) with a width of 15.0 mm, a depth of 150 mm, and a thickness of 0.15 mm was laminated on the surface (b). The double-faced tape was selected because it consists of a silicone adhesive, which can adhere well to the silicone dielectric layer. Moreover, the double-faced tape does not have a base film, such as PET, but consists only of adhesive; thus, it can follow expansion and shrinking. The silver-plated fiber (AGposs, Mitsufuji, Tokyo, Japan) was cut to 5.0 ± 0.5 mm in length (processed by Chubu-Pile, Aichi, Japan) and filled into a flocking device (Mini-flocker, Shin-Nissen, Osaka, Japan). The short fibers were released from the device, while 70 kV was applied between the sheet and the flocking device, and the surface of the double-faced tape was coated with silver-plated fibers (c). This process resulted in the formation of a conductive layer composed of silver-plated fibers. A precision balance (ME103E, Mettler Toledo, Tokyo, Japan) was used to measure the attached weight and to confirm the density of the silver-plated fibers flocked to the conductive layer. An optical microscope (DSX1000, Olympus, Tokyo, Japan) was used to observe the flocking of the silver-plated fibers. The sheet resistance of the conductive layer was evaluated using a four-terminal measurement device (MCP-T700, Nitto Seiko, Kanagawa, Japan). Thereafter, a copper tape (No. 8315, Teraoka Seisakusho, Tokyo, Japan) with a conductive adhesive layer was bonded to the edge of the conductive layer to form a mechanical terminal for use in the later soldering process. The same double-faced tape as in step (b) was fixed around the periphery of the conductive layer (d). A silicone sheet (SR300, Tigers Polymer, Osaka, Japan) with a thickness of 0.3 mm, cut out to a width of 35.4 mm and a depth of 150 mm, was laminated (e). The purpose of this lamination was to protect the conductive layer and provide mechanical strength to the sample. As shown in (f), the sample was flipped, and the opposite surface was laminated with double-faced tape in the same manner as in (b). Subsequently, the process from (c) to (e) was repeated to fabricate the capacitive sensor (g). As illustrated in the cross-sectional view (h), it consisted of a dielectric layer sandwiched between two conductive layers fabricated by flocking Ag-plated fibers.

The fabricated sensor was incorporated into a commercially available belt to provide wearability. The belt was made of nylon fiber and had hook-and-loop fasteners and hardware. The belt was cut into two pieces, and the end of the sensor was glued to each piece. An insulated nichrome wire was soldered to two terminals made of copper tape prepared during the fabrication of the sensor, making it possible to connect to the measurement device. The length of the nichrome wire was sufficiently long (5 m), such that it cannot interfere with human movement in the experiments described in Section 2.2.

The sensor was connected to an LCR meter (AX-221N, Adex Aile Co. Ltd., Kyoto, Japan), with $\pm \left(0.3\%+0.4\right)\;\mathrm{pF}$ accuracy, to measure the capacitance. The measurement conditions were an applied voltage of 2.0 V and a measurement frequency of 1.0 kHz. To understand the basic relationship between the amount of sensor elongation and the change in capacitance, we attached the sensor to a device that mechanically displaces in one direction (EMX-1000, Imada, Aichi, Japan) and quantitatively measured the amount of sensor elongation and the change in capacitance. As described in Section 2.2, the LCR meter outputs the capacitance value as an analog voltage to the optical motion capture system for synchronous measurement.

### 2.2. Experiments on Human Subject

**Figure 2:**
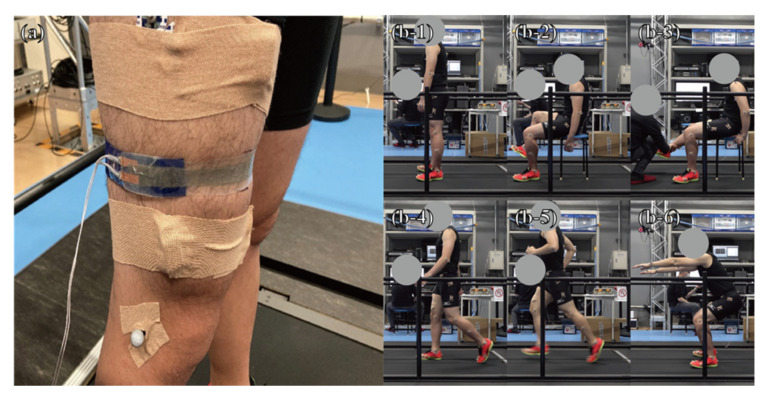
(**a**) Attachment of the DATSURYOKU sensor. (**b-1**) Standing position, (**b-2**) sitting position, (**b-3**) passive extension and flexion of knee joint, (**b-4**) walking, (**b-5**) running, and (**b-6**) squatting.

To validate the DATSURYOKU sensor developed in the previous section, we conducted experiments on a human subject using the DATSURYOKU sensor and conventional measurement systems to measure the muscle state in a multimodal manner (Figure 2). In this experiment, a participant stood, sat, walked, and ran on a treadmill with built-in force plates. His/her movement and ground reaction force (GRF) data were measured using an optical motion capture system installed around the treadmill and a force plate built into the treadmill, respectively. In addition, electromyography was used to measure muscle activity. The DATSURYOKU sensor was wired to the optical motion capture system via an LCR meter and measured the circumference data that was synchronized with data from other conventional measurement systems.

One healthy male, 29 years old, participated in this study. The subject was not injured at the time of data collection. The protocol of this study was approved by the review committee of the local research institute in accordance with the guidelines described in the Declaration of Helsinki (1983), and informed consent was obtained from the subject. A split-type treadmill with a built-in force plate (ITR5018-11C, Bertec, Columbus, OH, USA) was used for the measurements. Two force plates installed on the treadmill were used to measure the contact force between the participant and the floor. Each force plate measured the contact force and momentum in six axes at a rate of 1 kHz. In addition, the activities of the vastus lateralis (VL), rectus femoris (RF), vastus medialis (VM), biceps femoris long head (BFL), and biceps femoris short head (BFS) muscles of the right leg were recorded at a rate of 1 kHz using a wireless EMG system (picoEMG, Cometa Srl, Bareggio MI, Italy). A data acquisition device (USB-6218, National Instruments, Austin, TX, USA) and a motion capture system (Cortex-64 7.2.6.1828, Motion Analysis Co., Santa Rosa, CA, USA) were used to record three-dimensional marker positions and analog data, including contact forces and muscle activity data synchronously. The DATSURYOKU sensor was connected to a data acquisition device and a motion capture system via an LCR meter (AX-221N, Adex Aile Co., Ltd., Kyoto, Japan). The circumference was recorded using a data acquisition device and motion capture system, synchronized with data from other conventional measurement devices.

Here, the participant performed a single maximal voluntary co-contraction of the thigh muscles for 10 s in the static standing and sitting positions, and passive extension and flexion of the knee joint 5 times in the sitting position to monitor the characteristics of the sensor. Data during walking (4 km/h) and running (12 km/h) for 2 min on the treadmill were also collected during conscious co-contraction and neutral and conscious relaxation. In addition, a series of five maximal voluntary co-contractions in the sitting position and five squats were performed as movements with and without explicit co-contraction.

The recorder circumference data from the DATSURYOKU sensor were low-pass filtered at 10 Hz using a second-order Butterworth filter. The recorded GRF data were low-pass filtered at 50 Hz using a second-order zero-lag Butterworth filter [15]. The EMG data were high-pass filtered at 20 Hz using a second-order zero-lag Butterworth filter, that is full-wave rectified, and low-pass filtered at 10 Hz using a second-order Butterworth filter [16]. The activity of each muscle in each subject was normalized by the maximum isometric muscle contraction (MVC), which is the maximum activity of each muscle during co-contraction in the static standing and seated positions [17]. Data from standing and sitting positions were graphed synchronously, and data from walking and running were trimmed and normalized based on the gait cycle, with heel contact determined at the first frame when the vertical GRF was greater than 20 N [18], which was also checked using the motion data measured by the optical motion capture system. The mean and standard deviation for each % gait cycle were calculated.

## 3. Results

### 3.1. Evaluation of the DATSURYOKU Sensor

**Figure 3:**
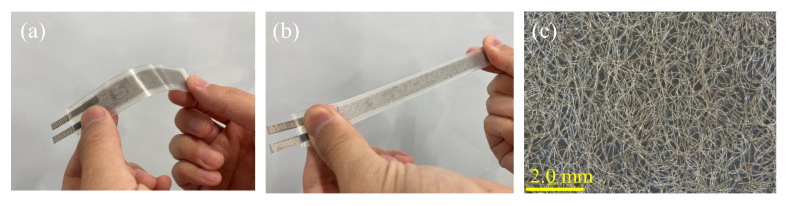
Appearances of the fabricated sensor ((**a**): non-stretched, (**b**): stretched) and a micrograph of the silver-plated fiber flocked in the conductive layer (**c**).

The appearance of the fabricated sensor is shown in Figure 3a,b. Owing to the flexibility of the silicone, the sensor can be easily stretched by hand. As both the silicone sheet and the silicone adhesive are transparent, the flocked layer of silver-plated fiber can be observed from the outside. Figure 3c shows the magnification of this layer under the microscope. It was confirmed that the silver-plated fibers were evenly spread over the entire adhesive area. The weight of the sample increased by 18 ± 2 mg owing to the flocking of the silver-plated fiber, as shown in Figure 1c. The weight density of the conductive film by the silver-plated fiber was 8.0 × 10^−3^ mg/mm^2^, because the area of the flocking was 15 mm wide and 150 mm depth. The sheet resistance of the conductive film measured by the four-terminal method was 1.2 ± 0.1 Ω/sq. This electrical resistance is comparable to that in our previous report [14]. Compared to conductive films on common flexible films, the resistance was lower than that of conductive oxides such as indium tin oxide, but higher than that of metals such as aluminum. This might be because the silver-plating layer on the surface of the fibers contributing to the conductivity was significantly thin (approximately 100 nm) [19]; moreover, they physically contacted each other to form a conductive path. This conductivity was an acceptable performance of the silver-plated fiber, and the resistance was sufficiently low to allow capacitive sensors to operate.

**Figure 4:**
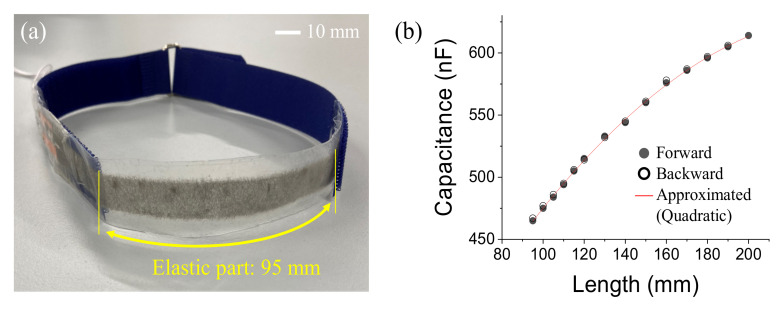
Appearances of the sensor integrated into a belt (**a**) and the relationship between the capacitance of the sensor and the elastic part’s length (**b**).

Figure 4a shows the appearance of the sensor, which was integrated into a belt to provide wearability. As depicted in Figure 4a, the length of the elastic part of the sensor is 95 mm because both ends of the sensor are bonded with a low-elastic nylon belt. Figure 4b shows the results of measuring the capacitance of the sensor while stretching it step by step. The horizontal and vertical axes correspond to the length of the elastic part of the sensor, and to the capacitance of the sensor, respectively. From the initial length of 95 mm, 465 nF is obtained. As the sensor is stretched, the capacitance increases nonlinearly, and 606 nF can be observed from a length of 200 mm, which is the most stretched state in this experiment. The capacitance of the sensor was stably measured even when the sensor was stretched by approximately 110% from 95 to 200 mm in length. Figure 4b shows the results of the stretching (forward) and shrinking (backward) processes of the sensor. As these plots overlap, it can be confirmed that the capacitance of the sensor has no hysteresis against mechanical expansion and compression. From the quadratic approximation of the forward result, the red line in Figure 4b was obtained with a 99.84% agreement. The length of the elastic part of the sensor (*L* (mm)) is expressed in terms of its capacitance (*C* (nF)), which results in Equation (1). From these results, we succeeded in developing a stretchable sensor that measures the change in length from the change in capacitance with good wearability.

(1) $$L=0.00223C^{2}-1.72592C+416.9272$$

### 3.2. Evaluation of Human Subject Experiments

**Figure 5:**
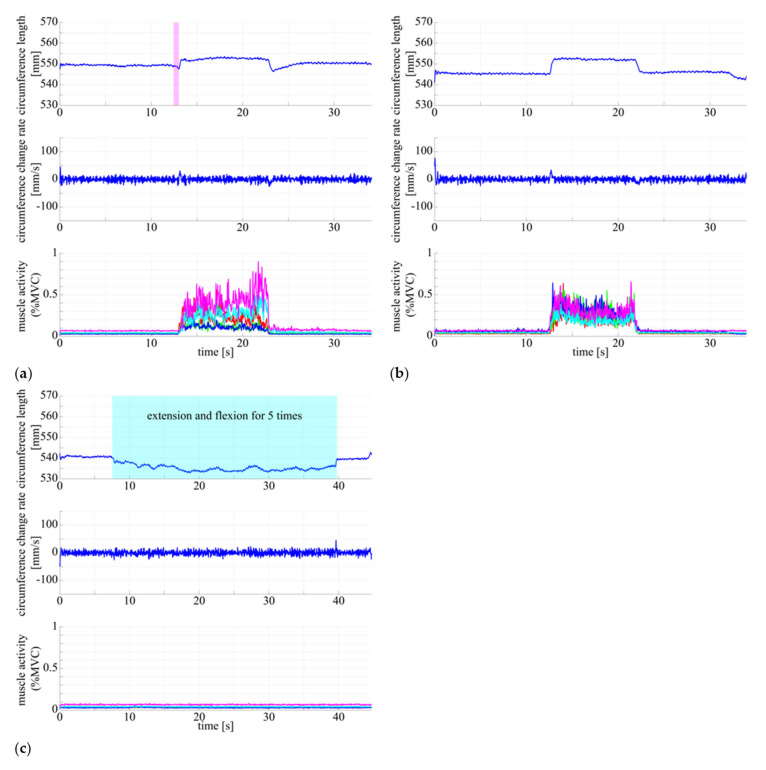
Circumference length, velocity, and muscle activity of (**a**) co-contraction during static standing position, (**b**) co-contraction during sitting position, and (**c**) relaxation during passive extension and flexion of knee joint (5 times during the middle of sequence). The horizontal axis represents time (s) and the vertical axis represents the stretch length (mm) in top, stretch velocity (mm/s) in middle, and muscle activity normalized by MVC in bottom. The red and green lines represent vastus lateralis and vastus medialis, respectively; the blue, cyan, and magenta lines represent rectus femoris, biceps femoris long head, and bicep femoris short head, respectively.

**Figure 6:**
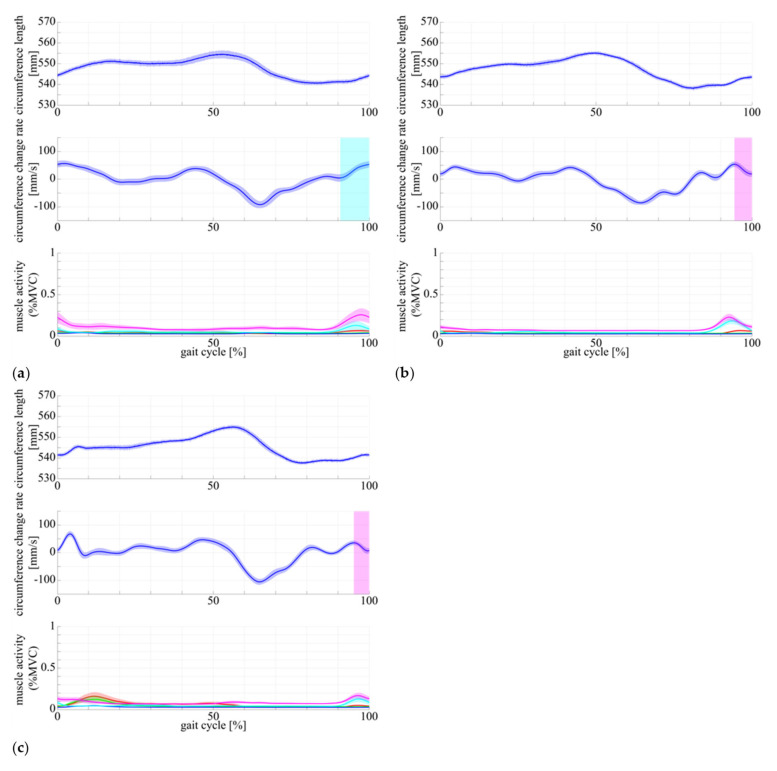
Stretch length, velocity, and muscle activity of walking with (**a**) conscious muscle co-contraction, (**b**) neutral, and (**c**) conscious muscle relaxation. The horizontal axis represents the gait cycle (%), and the vertical axis represents the stretch length (mm) in top, stretch velocity (mm/s) in middle, and muscle activity normalized by MVC in bottom. Each color of line represents same item as the one in Figure 5.

**Figure 7:**
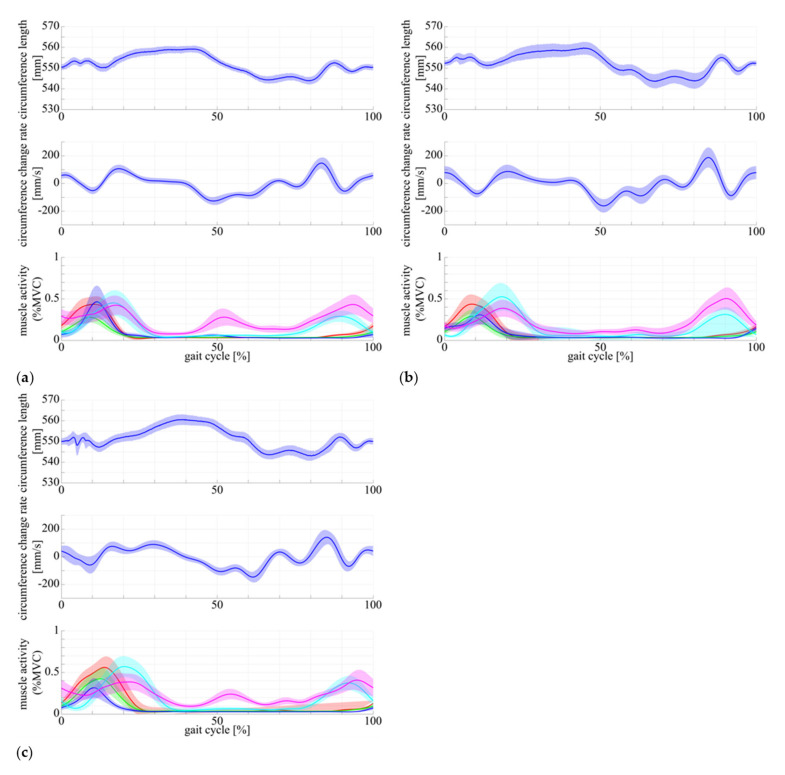
Stretch length, velocity, and muscle activity of running with (**a**) conscious muscle co-contraction, (**b**) neutral, and (**c**) conscious muscle relaxation. The horizontal axis represents the gait cycle (%), and the vertical axis represents the stretch length (mm) in top, stretch velocity (mm/s) in middle, and muscle activity normalized by MVC in bottom. Each color of line represents same item as the one in Figure 5.

Here, we outline the results of multimodal measurement of muscle state in human subject using the DATSURYOKU sensor and conventional measurement devices. In each graph in Figure 5, the horizontal axis represents time (s), and the vertical axis represents circumference (mm), rate of change of circumference (mm/s), and muscle activity (%MVC). The red, green, blue, cyan, and magenta lines represent the vastus lateralis, rectus femoris, vastus medialis, biceps femoris long head, and biceps femoris short head, respectively. Figure 5 show the results of measurements of circumference length, velocity, and muscle activity during (a) co-contraction of all muscles in the static standing position, (b) co-contraction of all muscles in the sitting position, and (c) passive extension and flexion of the knee joint in the sitting position. Figure 6 and Figure 7 show the muscle state during walking and running, respectively. In each graph, the horizontal axis is the percentage of the gait cycle (%), and the vertical axis is the circumference (mm), rate of change of circumference (mm/s), and muscle activity (%MVC). In each graph, the solid line represents the mean value over the entire gait cycle, and the semi-transparent area represents the standard deviation. The colored lines of the muscle activities are the same as those shown in Figure 5. Table 1 shows the average amplitudes of circumference from the natural length in a gait cycle, the average standard deviations between cycles, and their coefficient of variation. Table 2 shows the average amplitudes of muscle activities (%MVC) in a gait cycle, average standard deviations between cycles, and their coefficient of variation.

**Figure 8:**
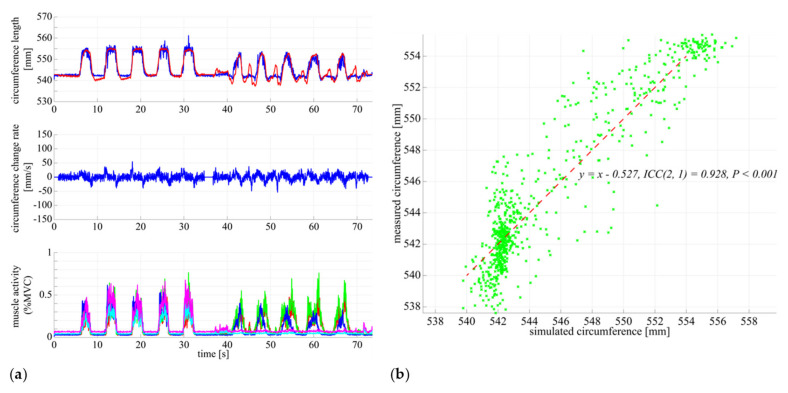
Relationship between EMG and stretch length. (**a**) Measured stretch length, velocity, muscle activity and estimated stretch length during the muscle co-contraction at static standing posture and squat motion. The horizontal axis represents time (s) and the vertical axis represents the stretch length (mm) in top, stretch velocity (mm/s) in middle, and muscle activity normalized by MVC in bottom. The red line represents the measured stretch length and the blue one represents the estimated one. (**b**) Relationship between the simulated and measured stretch lengths. The horizontal axis represents the simulated stretch length (mm) and the vertical axis represents the simulated stretch length (mm). The green cross represents the data sampled in 10 Hz and the red line represents the regression line between these two data.

Figure 8 shows the results of modeling the relationship between muscle activity and circumference. We assumed that the circumference can be expressed as a quadratic form of the involved muscle activities, and then we optimized the coefficients by least square problem to minimize the following evaluation function Equation (3) in the measured data:

(2) $$\hat{L}\left(t\right)=\overset{5}{\underset{i=1}{\sum }}(w_{i1}A_{i}\left(t\right)+\;w_{i2}A_{i}^{2}\left(t\right))+b$$

(3) $$E=\overset{T}{\underset{t=1}{\sum }}{\left(L\left(t\right)-\hat{L}\left(t\right)\right)}^{2},$$

where $\hat{L}\left(t\right)$ represents the estimated circumference, $A_{i}\left(t\right)$ represents the *i*-th muscle activity (i=1⋯5), $w_{i1}$ and $w_{i2}$ represent the coefficients for the *i*-th muscle, $L\left(t\right)$ represents the measured circumference, and T represents the frame number of the measured data. Figure 8a in the top panel shows the measured circumference and the time series of the circumference calculated using Equation (2), and Figure 8b shows the relationship between them. The interclass correlation coefficient (ICC(2,1)) was used to assess the relationship between the measured and simulated stretch lengths. In Figure 8a, the horizontal axis represents time (s), and the vertical axis represents the circumference (mm), rate of change of the circumference (mm/s), and muscle potential (%MVC). In Figure 8b, the vertical axis represents the circumference measured by the DATSURYOKU sensor, the horizontal axis represents the estimated circumference calculated from Equation (2), and the red solid line represents the regression line. From the experimental results, we can point out the following:

- As shown in Figure 5a,b, the circumference increases in synchronization with the muscle activities. The same pattern of circumference change occurred regardless of standing or sitting posture. In the passive extension and flexion of the knee joint in the sitting position, no muscle activity was observed, and no increase in circumference was observed.
- In Figure 6 and Figure 7, muscle activity occurs in synchronization with the walking and running gait cycle, and a pattern of circumference change occurs correspondingly. In addition, there is a difference in the patterns of (a) conscious co-contraction, (b) neutral, and (c) conscious relaxation.
- In Figure 8, the model obtained by the least square problem well represents the relationship between muscle activities and circumference for both co-contraction in the sitting position and squatting, in which no muscle co-contraction was observed. The mean square error between the measured circumference and the circumference calculated from the EMG potential was 1.83 mm. The regression line between the two circumferences was $y=x-0.527,\;ICC\left(2,\;1\right)=0.928,\;P<0.001$, indicating a high interclass correlation.

## 4. Discussion

The human subject experiments using the DATSURYOKU sensor and conventional measurement devices suggest the following:

- Figure 5a,b show that the DATSURYOKU sensor can measure changes in circumference in response to muscle activity. The pattern of the change is similar to the pattern of the muscle activities regardless of posture, such as standing or sitting. In the standing position, the circumference of the muscle decreases at the beginning of the transition from standing to co-contraction and then increase after the decrease (magenta section in Figure 5a), which may be because of the temporary muscle relaxation before co-contraction. These results show that it is possible to measure the muscle tension and co-contraction that do not appear in the motion by measuring the circumference. In Figure 5c, the circumference decreases during passive extension and flexion of the knee joint, which may be because of passive muscle relaxation caused by the shortening of the muscle length during passive knee joint extension. The circumference is believed to decrease owing to the tightening force applied by the sensor. These results indicate that the DATSURYOKU sensor can measure muscle tension and co-contraction that do not appear in motion, as well as passive deformation of muscles that do not appear in muscle activity.
- From Figure 6 and Figure 7, muscle activity occurs in synchronization with the walking and running cycles, and the pattern of circumference change occurs correspondingly. The coefficient of variation, which is the average standard deviation between gait cycles divided by the average amplitude of one cycle, was small (8.19% for walking and 1.68% for running), whereas the muscle activity (%MVC) was 1.56% and 2.90% for walking and running, respectively. This result indicates that the DATSURYOKU sensor can stably measure the circumference, which represents the muscle state. The difference in the patterns between (a) conscious co-contraction, (b) neutral, and (c) conscious relaxation is that in (a) the muscle tension increases toward heel contact (positive velocity of circumference change, cyan sections in Figure 6a, while in (b) and (c) the muscle tension decreases and then increases (magenta sections in Figure 6b,c). In addition, the amplitudes within a cycle (a) < (b) < (c) in Table 1 indicate that the muscle tends to always exert itself in (a), whereas in (c), the muscle activity is well modulated. This result suggests that by observing the circumference pattern of the DATSURYOKU sensor, it is possible to detect persistent muscle tension, etc., and to estimate the risk of injury, such as load on the knee joint.
- Figure 8 shows that the model obtained by the least square problem well represents the relationship between muscle potential and circumference for both co-contraction and squatting in the sitting position. The regression line between them is $y=x-0.527,\;ICC\left(2,\;1\right)=0.928,\;P<0.001$, which shows a high interclass correlation. This indicates that the circumference represents the muscle state well both with and without co-contraction, and that the DATSURYOKU sensor is suitable for measuring excessive muscle tension and co-contraction, which is the aim of this paper. The root mean square error between the measured circumference and the circumference calculated from the muscle activities is 1.83 mm. Considering that the amplitude in the walking cycle is 1.62 mm on average of (a), (b), and (c), and that in the running cycle is 1.63 mm on average of (a), (b), and (c), the obtained model estimates the muscle state with high accuracy. Figure 8a shows that the error becomes larger when the circumference decreases. In Figure 8b, the distribution of points spreads vertically where the circumference becomes negative. This result reveals that the circumference measured by the DATSURYOKU sensor shows passive deformation, while the muscle activity becomes zero. These results indicate that the DATSURYOKU sensor can measure muscle passive deformation that does not appear in muscle activity.

Table 3 summarizes the pros and cons comparing the DATSURYOKU sensor with EMG.

## 5. Conclusions

In this study, we developed a DATSURYOKU sensor that has the potential to be used to measure the circumference for cross-modal measurement of muscle state to routinely measure excessive muscle tension and co-contraction in daily life, which can be caused by poor performance, failure, and injury in sports, as well as malfunctions and diseases in daily activities, such as contractures and tremors. The DATSURYOKU sensor is a belt-shaped capacitive sensor that detects the circumference of the applied part. The stretchable electrode formed by the flocking of silver-plated fibers was effective in achieving the sensor function, which showed a continuous and clear capacitance change even for 110% expansion. We measured the muscle state in a multimodal manner, with a root mean square error of 1.83 mm and interclass correlation coefficient of $ICC\left(2,\;1\right)=0.928,\;P<0.001$ between the measured circumference and the one estimated in quadratic form of muscle activities. The decrease in the circumference during muscle relaxation indicates passive deformation caused by an external force. These results indicate that the DATSURYOKU sensor can measure muscle tension and co-contraction that do not appear in motion, as well as passive muscle deformation that does not appear in muscle activity.

The limitation of this study is the evaluation of the sensor. We modeled the sensor characteristics and showed the possibility of measuring excessive muscle tension and co-contraction with small number of evaluations. We need additional precise sensor evaluations to comprehensively demonstrate the performance of the sensor and use it for the daily routine muscle state measurements, including thermo stability, deformation reading rates, resistance to other types of deformation (pressure), and accurate comparison with EMG. Another limitation is that the DATSURYOKU sensor measures the effect of all muscles involved in the circumstance and cannot measure the independent muscle activity. We would solve this limitation by considering the musculoskeletal model and estimating this identical muscle activity by solving the mathematical optimization of somatosensory information estimation. Several directions remain for future work. Current DATSURYOKU sensors are meant for measurement in laboratory. We are working on the development of a wireless and wearable measurement with Bluetooth connection and on-tip data acquisition for the daily routine measurement. Another direction for the DATSURYOKU sensor is to realize the large-scale muscle state dataset from its daily routine measurement. We plan to apply machine learning techniques for injury risk estimation and sports performance improvement. Another direction is to apply the DATSURYOKU sensor only on the young subject. With further experimentation, we plan to validate this sensor for elderlies and children whose muscle properties are different from young people.

## Figures and Tables

**Table 1:** Statistical analysis of circumference during walking and running with (a, d) conscious muscle co-contraction, (b, e) neutral, and (c, f) conscious muscle relaxation.

	Walk			Run		
	(a)	(b)	(c)	(d)	(e)	(f)
Average amplitude in a cycle (mm)	130	130	128	135	135	134
Average deviation between cycles (mm)	1.31	0.924	0.963	1.72	2.69	2.38
Coefficient of variation (%)	0.998	0.710	0.751	1.28	1.98	1.77

**Table 2:** Statistical analysis of muscle activity (%MVC) during walking and running with (a, d) conscious muscle co-contraction, (b, e) neutral, and (c, f) conscious muscle relaxation.

	Walk			Run		
	(a)	(b)	(c)	(d)	(e)	(f)
Average amplitude in a cycle	0.0554	0.0487	0.0559	0.128	0.123	0.140
Average deviation between cycles	0.0120	0.00561	0.00977	0.0364	0.0412	0.0390
Coefficient of variation (%)	19.2	10.8	16.9	27.3	32.1	27.7

**Table 3:** Pros and cons comparing the DATSURYOKU sensor with EMG.

	EMG	DATSURYOKU Sensor
measurable detailedness	independent muscle activity	effect of all muscles involved in circumstance
measurable characteristics	muscle activity	muscle status that involves activity and passive deformation
stability	electric contact impedance changes because of sweat and motion	physical displacement affects
battery	high sampling consumes power	low sampling
calibration	need to measure MVC at each attachment.	need to calibrate once for one sensor.

## Data Availability

The data presented in this study are available on request from the corresponding author. The data are not publicly available due to privacy restrictions.
